# Rapid maxillary expansion and obstructive sleep apnea: A review and meta-analysis

**DOI:** 10.4317/medoral.21073

**Published:** 2016-03-31

**Authors:** Almiro-José Machado-Júnior, Edilson Zancanella, Agrício-Nubiato Crespo

**Affiliations:** 1 DDS, PhD. Discipline of Otorhinolaryngology, Unicamp (Campinas State University) -São Paulo - Brazil; 2 MD, PhD. Discipline of Otorhinolaryngology, Unicamp (Campinas State University) - São Paulo - Brazil; 3 MD, PhD (Full professor). Discipline of Otorhinolaryngology, Unicamp (Campinas State University) - São Paulo - Brazil

## Abstract

**Background:**

OSAS during childhood leads to significant physical and neuropsychomotor impairment. Thus, it needs to be recognized and treated early in order to avoid or attenuate the chronic problems associated with OSAS, which are deleterious to a child’s development. Adenotonsillectomy and, in select cases, continuous positive airwaypressure (CPAP) have been the preferred treatments for OSAS in children, and yet they are ineffective at fully ameliorating the disease. Minimally invasive treatments have recently been proposed, comprising intra-oral and extra-oral devices as well as speech therapy. Objetive: to conduct a meta-analysis on studies from around the world that used rapid maxillary expansion (RME) to treat OSAS in children.

**Material and Methods:**

We performed a meta-analysis of studies using RME for OSA treatment in children. A literature survey was conductedusing PubMed and Medline for English articles published up to December 2014 with the following descriptors: Sleep Apnea, Obstructive, Children, Treatment, Orthodontic, Othopaedic, Maxillaryexpansion. Studies were included in the meta-analysisif they were case-controlled studies, randomized, and involved non-syndromic children aged 0 to 12years old diagnosed with OSA by the polysomnography apnea-hypopnea index (AHI) before and after the intervention, submitted RME only.

**Results:**

In all, 10 articles conformed to the inclusion criteria and were included in this meta-analysis. The total sample size across all these articles was 215 children, having a mean age of 6.7 years,of whom58.6%were male. The mean AHI during the follow-up was -6.86 (*p* <0.0001).

**Conclusions:**

We concluded that rapid maxillary expansion (RME) in children with OSAS appears to be an effective treatment for this syndrome. Further randomized clinical studies are needed to determine the effectiveness of RME in adults.

**Key words:**Rapid maxillary expansión, obstructive sleep apnea, meta-analysis.

## Introduction

Recently, great advancements have taken place in sleep disorder research, among which, the characterization of obstructive sleep apnea syndrome (OSAS) is perhaps the most significant (1-3). This has revealed the complexity of OSAS and demonstrates a need for multidisciplinary interrelations spanning various healthcare fields. OSAS is a chronic evolutive disease with high morbidity and mortality rates (4-7). Disease etiology comprises a polymorphous set of symptoms ranging from snoring to excessive daytime somnolence, co-presenting with severe general hemodynamic, neurological and behavioral repercussions (8-11).

Many studies have aimed to define the anatomical abnormalities that predispose patients to OSAS and detail various tests and treatments for OSAS in adult populations (12-15). However, there are only a small number of studies on OSAS in children, although it might be possible to diagnose and treat the disease early in life (1-5). Recent studies have correlated orofacial dysfunctions with OSAS in children (16-19).

OSAS during childhood leads to significant physical and neuropsychomotor impairment. Thus, it needs to be recognized and treated early in order to avoid or attenuate the chronic problems associated with OSAS, which are deleterious to a child’s development (2-7). Adenotonsillectomy and, in select cases, continuous positive airway pressure (CPAP) have been the preferred treatments for OSAS in children, and yet they are ineffective at fully ameliorating the disease (1-9). Minimally invasive treatments have recently been proposed, comprising intra-oral devices (19-27) as well as speech therapy (28). Among the available intra-oral devices, rapid maxillary expansion (RME) has been used to treat OSAS among children (19-27) . However only limited studies have evaluated this treatment for its efficacy in ameliorating OSAS symptoms (19-27). Therefore , there is no consensus about the benefits of using RME to treat OSAS (1,2,19-27). The present study aimed to conduct a meta-analysis on studies from around the world that used RME to treat OSAS in children.

## Material and Methods

A bibliographic search for all articles published in English up to December, 2014 was conducted using PubMed and Medline with the following search terms:Sleep Apnea, Obstructive, Children,Treatment, Orthodontic, Orthopedic and Maxillary Expansion.

**Figure 1:**
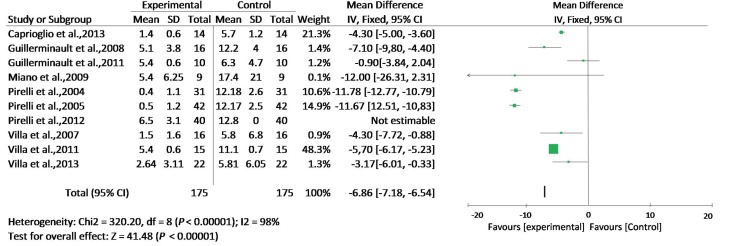
Selected studies to conduct the meta-analysis.

The inclusion criteria restricted the search to studies having the following characteristics: randomized trials, case-control or cohort studies,and studies on non-syndromic children between the ages of 0 and 12 years with a diagnosis of OSAS from polysomnography, who underwent RME with polysomnography afterwards, and for whom an apnea-hypopnea index (AHI) score was available both before and after RME (Fig. 1). After selecting studies that met the inclusion criteria, data on sex, age, and AHI before and after RME were compiled. Treatment effects were combined by means of a meta-analysis using the random-effects method (29,30).

The means were grouped using the weighted means method, according to the weight that each study represented, as assessed according to the sample size and variance presented in the study. The differences were then compared using the weighted means method (weightedmeans score). Comparisons were made using the mean, standard deviation, and 95% confidence interval, as well as the difference between the time in question and the baseline were obtained. Thus, negative means indicated that there was a decrease in the mean values. Confidence intervals that did not pass through the value zero indicated a significant effect (*p* < 0.05).The software used for the analysis was comprehensive meta-analysis version 2.2. The significance level was 5%.

## Results

**Figure 2:**
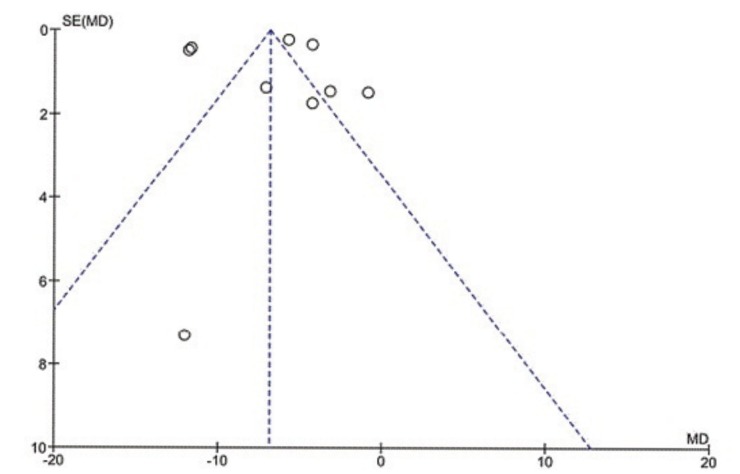
The mean AHI during the follow-up evaluation.

Ten articles that conformed to the inclusion criteria were used in this meta-analysis ( Table 1, Figs. 1,2). The total size of the sample in these articles was 215 children, with a mean age of 6.7 years ( Table 1) and 58.6% of subjects were male ( Table 1). The mean AHI during the follow-up evaluation was -6.86 (*p* <0.0001) (Fig. 2).

**Table 1:**
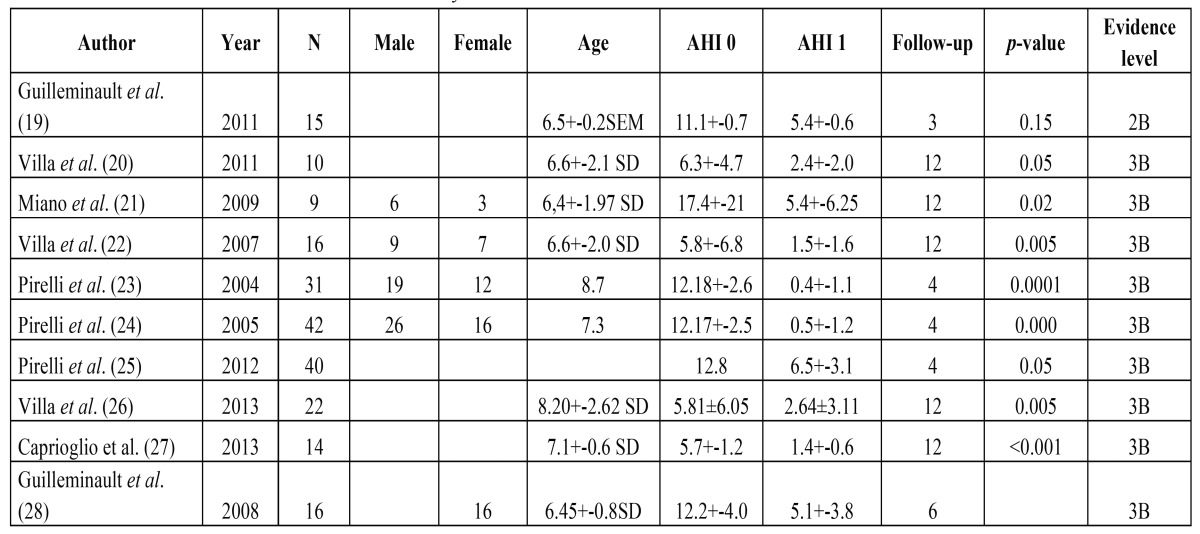
Selected studies to conduct the meta-analysis.

To assess whether the selected studies were capable of being compiled, we performed a heterogeneity test, yielding an inconsistency between studies: Chi² = 320.20, df = 8 (*p*<0.00001); I² = 98%. To Higgins *et al.,* largest heterogeneities that 75% is considered high when performing the meta-analysis is questionable (30) (Fig. 2).

## Discussion

The results of this meta-analysis show that a limited number of studies used RME in treating childhood OSA. In addition, there were no randomized controlled trials and high heterogeneity existed between studies. However, studies included in this meta-analysis indicate that there is decrease in AHI after maxillary expansion in children with OSAS. Additionally, these studies suggest that the AHI decline is maintained as indicated by follow-up tests ranging from 3 months to 14 years (seven years after ERM) (3).

Several theories attempt to explain why there is a measurable decrease in AHI after RME, in children with OSAS. It is believed that RME decreases nasal resistance and facilitates the passage of air through the nose. In addition to improving the quality of nasal respiration, RME increases the maxillary dental arch and thus improves the position of the tongue enabling propersealing of the lips when the mouth is closed. It also indirectly increases the oropharyngeal space (28). These effects of RME may contribute towards diminishing OSAS in children. However, further studies are needed in order to fully test this hypothesis.

Villa *et al.* (2007) observed that 78.5% of the children that they studied presented with enlarged tonsils and that after RME, the tonsils shrank. They emphasized that this decrease was relative, since there were increases in the size of adjacent structures (22). This was also observed by Pirelli *et al.* (2004), who reported that after RME, there was an increase in the oropharyngeal space and modification of the position of the tongue (23).

These observations led us to ask whether RME is capable of increasing the oropharyngeal space and whether it wouldindirectly affect the size of the tonsils. In four of these six studies, adenotonsillectomy was not performed, yet the results obtained were close to those of the studies in which it was. However, the studies in which RME was the sole treatment excluded children who had undergone a surgical treatment for OSAS (19-22). This may have introduced selection bias into the studies and implied that children with a lower degree of tonsil enlargement were selected. On the other hand, in one of these studies, it was observed that RME produced a decrease in tonsil volume (23). Further studies are needed in order to evaluate the limits for RME treatment of OSAS in children with large-volume tonsils and adenoids.

Another important factor emerging from a meta-analysis of these studies was that in addition to the AHI, children also co-presented symptoms associated with sleep quality, cognitive factors, somnolence and irritability (19-23). There was a trend among all these articles and the present meta-analysis suggest that the hypothesis that RME diminishes the AHI of children with OSAS. However, these studies were not unanimous in supporting the hypothesis that there were improvements in non-respiratory factors associated with OSAS. Further controlled studies are required in order to test this hypothesis.

A relationship between OSAS and mouth-breathing has been demonstrated in children other studies (22). These studies also observed that after RME, there is a substantial decrease in mouth-breathing of treated children. In children with nasal obstruction, RME not only reduces nasal obstruction but also raises tongue posture and enlarges the pharyngeal airway (19). Were commend conducting future studies focusing the relationship between OSAS and mouth-breathing in children.

We conclude that rapid maxillary expansion in children with OSAS appears to be another effective treatment for this syndrome. Further randomized clinical studies are needed to assess whether this treatments efficacy is retained throughout adulthood.
